# Pharmacological inhibition of neuropeptide Y receptors Y1 and Y5 reduces hypoxic breast cancer migration, proliferation, and signaling

**DOI:** 10.1186/s12885-023-10993-1

**Published:** 2023-06-01

**Authors:** Sydney A. Pascetta, Sarah M. Kirsh, Makenna Cameron, James Uniacke

**Affiliations:** Department of Molecular and Cellular Biology, University of Guelph, 50 Stone Road East, Guelph, ON N1G 2W1 Canada

**Keywords:** Hypoxia, GPCR, NPY, Breast cancer, Neuropeptide

## Abstract

**Background:**

Neuropeptide Y (NPY) is an abundant neurohormone in human breast carcinomas that acts on a class of G-protein coupled receptors, of which NPY1R and NPY5R are the most highly expressed. This abundance is exploited for cancer imaging, but there is interest in pharmacological inhibition of the NPYRs to interrogate their functional relevance in breast cancer. We previously reported that NPY1R and NPY5R mRNA abundance is increased by hypoxia inducible factors, which sensitizes these receptors to NPY stimulation leading to enhanced migration and proliferation.

**Methods/Results:**

Here, we measured the effects of NPY1R and NPY5R antagonists in normoxia and hypoxia on migration, proliferation, invasion, and signaling in 2D and 3D models of breast cancer cell lines MDA-MB-231 and MCF7. Antagonizing NPY1R and/or NPY5R in hypoxia compared to normoxia more greatly reduced MAPK signaling, cell proliferation, cell migration and invasion, and spheroid growth and invasion. The estrogen receptor positive MCF7 cells were significantly less invasive in 3D spheres when NPY5R was specifically inhibited. There were some discrepancies in the responses of each cell line to the isoform-specific antagonists and oxygen availability, therefore further investigations are required to dissect the intricacies of NPYR signaling dynamics. In human breast tumor tissue, we show via immunofluorescence that NPY5R protein levels and colocalization with hypoxia correlate with advanced cancer, and NPY1R protein correlates with adverse outcomes.

**Conclusions:**

Antagonizing the NPYRs has been implicated as a treatment for a wide variety of diseases. Therefore, these antagonists may aid in the development of novel cancer therapeutics and patient-based treatment plans.

**Supplementary Information:**

The online version contains supplementary material available at 10.1186/s12885-023-10993-1.

## Introduction

Neuropeptide Y (NPY) is a 36 amino acid peptide amide named for its terminal tyrosine [1, 2]. NPY is produced in the central and peripheral nervous system and is the most abundant neuropeptide in the brain and spinal cord [3]. NPY acts on six G-protein coupled receptors (GPCRs) named NPY receptors (NPYRs). Most research has focused on NPY1R, NPY2R, and NPY5R because they are the most highly expressed and functionally relevant NPYR subtypes in humans [3, 4]. NPY1R and NPY5R expression is high in several types of tumors, such as ovarian, prostate, breast, and neural crest relative to normal tissue [3, 5–9]. Breast cancer in particular has come to the forefront because of its high frequency of NPYR overexpression and density compared with all other NPYR-positive tumors [9]. This characteristic has been exploited to develop chemically modified analogs of NPY that are being explored in breast cancer imaging and diagnosis [10–12]. When examining NPYR expression in breast carcinomas, only 24% are positive for NPY2R compared to 85% for NPY1R. Furthermore, breast cancer cell lines such as MDA-MB-231 and MCF7 have elevated levels of NPY1R and NPY5R [3, 4]. NPY1R and NPY5R stimulation promotes cellular proliferation, migration, and angiogenesis in breast cancer models [4, 13, 14]. Since breast tissue is highly innervated by the sympathetic nervous system and provided with a large supply of NPY ligand, it can be the perfect storm for the constitutive signaling of this pathway. Therefore, employing NPYR antagonists in the context of the tumor microenvironment could be a viable strategy in breast cancer therapy.

Hypoxia is a common feature of the tumor microenvironment that can lead to chemo- and radiation therapy resistance and promoting metastasis [15]. As solid tumors grow, their leaky vasculature provide an insufficient supply of oxygen to often hyper-consuming cancer cells. The cellular response to hypoxia is primarily driven by the hypoxia inducible transcription factors (HIFs) that induce an array of genes including vascular endothelial growth factor and angiopoietin-2 [16]. HIF-1 is a key regulator for glycolysis and pH regulation whereas HIF-2 is involved in proliferation and differentiation [17, 18]. Recently, our group demonstrated that NPY1R and NPY5R mRNA abundance is induced by the HIFs, sensitizing hypoxic cells to NPY-stimulated motility and proliferation in MCF7 and MDA-MB-231 breast cancer cells [19]. Simultaneously, signaling through the mitogen activated protein kinase (MAPK)/ERK pathway was induced more rapidly and potently upon NPY5R stimulation in hypoxic cells relative to normoxic cells. This caused hypoxic breast cancer cells to proliferate and migrate more than their normoxic counterparts. Therefore, hypoxia contributes to NPYR hyperactivity by increasing receptor production.

Recently, modulation of NPYRs has been implicated as a treatment for a wide range of diseases such as obesity, mood disorders, pain, and cancers [20]. The overexpression of NPY and its role in cancer progression could translate into cancer therapeutics, specifically through the use of NPYR antagonists, which have shown promising results for other diseases [21]. Preliminary studies on the use of NPYR antagonists as cancer treatments has also shown potential, making this a promising area of research. Further evidence on NPYR antagonism in the progression of cancer, especially in the context of hypoxic cell vulnerability, would shed valuable insight for the future of this potential therapeutic strategy.

Here we investigate the effect of antagonizing NPY1R and NPY5R isoforms on MAPK signaling, cell migration, cell proliferation and invasion, in 2D and 3D models of hypoxic and normoxic MCF7 and MDA-MB-231 breast cancer cell lines. These cell lines are used here as a continuation of our previous study showing they are more sensitive to NPY stimulation in hypoxia [19]. Further, these cell lines are each models of two different cancer subtypes that can provide insight into potential genetic differences in their susceptibility to NPYR antagonists. MCF7 is an estrogen receptor-positive model of the luminal A subtype and MDA-MB-231 is a triple-negative breast cancer (TNBC) basal-like subtype [22]. MAPK signaling was more greatly reduced in hypoxia in both cell lines when isoform-specific agonists were used in combination with antagonists. Only hypoxic MDA-MB-231 cell proliferation could be antagonized by NPYR inhibitors. Cell migration in MDA-MB-231 cells was only antagonized in normoxia, while hypoxia improved the effect of the antagonists in MCF7 only when stimulated with the general NPY agonist. Cell invasion was mostly repressed by antagonizing NPY5R in both cell lines, but hypoxia improved the effect of the Y5 antagonist when MCF7 cells were stimulated with the general NPY agonist. Spheroid growth, but not invasion, was repressed in MDA-MB-231 with NPYR antagonists, while MCF7 spheroid growth and invasion were both repressed specifically with the NPY5R antagonist. In human breast tumor tissue, we show that high NPY5R levels correlated with advanced stage cancer, metastasis, and poorly differentiated cells. Further, higher NPY1R levels correlated with poor patient outcomes such as death and progression-free survival. We show that antagonizing the NPYRs increased their own mRNA abundance in hypoxic spheroids. We observed some differences between cell lines and in response to oxygen, highlighting that more studies are required to decipher the complex signaling dynamics of the NPYRs in the tumor microenvironment. This study should help inform the future development of NPYR antagonists in breast cancer therapy and patient-based treatment plans based on NPYR levels.

## Methods

### Cell culture and reagents

MDA-MB-231 and MCF7 cells were obtained from the American Type Culture Collection and maintained in Dulbecco’s Modified Eagle Medium (DMEM) supplemented with 10% fetal bovine serum, as suggested. They were maintained mycoplasma free in a humidified chamber (5% CO_2_, and 37˚C). Cells were introduced to and maintained in hypoxia by incubating them in a HypOxystation H35 workstation (HypOxygen) at 1% O_2_, 5% CO_2_, and 37˚C. Cells were treated with NPYR agonists (Tocris):1 × 10^− 9^ M NPY (Cat#1153), 1 × 10^− 8^ M NPY1R-specific (Cat#1176), or 1 × 10^− 8^ M NPY5R-specific (Cat#1365), and NPYR antagonists (Tocris): 1 × 10^− 6^ M NPY1R (BIBP 3226, Cat #2707), 1 × 10^− 5^ M NPY5R (L-152, Cat# 1382) For spheroid experiments, BIBP 3226 and L-152 were used at 1 × 10^− 5^ M and 1 × 10^− 4^ M, respectively. Cells were pre-incubated with antagonists for 30 min prior to agonist exposure.

### pERK assay

7,250 MDA-MB-231 or 8,700 MCF7 cells were seeded in a black bottomed 96-well plate and exposed to serum-reduced media for 24 h in normoxia or hypoxia. Media was then replaced with serum-reduced media (0.5% FBS) with or without antagonist. After 30 min, additional serum-reduced media was added with or without agonist. Cells were then lysed after a period of 5, 15, and 30 min. Cells were then fixed and pERK/ERK was measured using fluorescence-based ELISA according to manufacture instructions (BioAssay Systems, EERK-100).

### Cell migration assay

Transwell migration assays were used to determine cell chemotaxis under given pharmacological conditions. Cells were given serum reduced (0.5% FBS) media for 24 h, then seeded at a density of 75,000 (MDA-MB-231) or 130,000 cells (MCF7) in the upper chamber of 12 well inserts with 8 μm pores (BD Biosciences). These cells were seeded in serum-reduced media in the presence or absence of antagonist. After 30 min, serum-reduced media with or without agonist was added to the bottom chamber. Cells were then exposed to 22 h (MDA-MB-231) or 24 h (MCF7) of normoxia or hypoxia. The near serum-starvation and end-point of ≤ 24 h was done to reduce the potential of cell proliferation to contribute to cell migration and invasion. Non-migrated cells were removed from the upper side of the inserts with a cotton swab and migrated cells were fixed in methanol and stained with Hoechst. Membranes were excised, mounted on slides, and imaged on a Nikon Eclipse Ti Microscope. Migrated cells were quantified using Fiji for ImageJ. A threshold was first determined that accurately highlighted migrated cells. A watershed mask was then applied to segregate adjacent cells. Parameters of circularity and size were applied, and number of particles was counted by ImageJ. Number of cells migrated were then compared to controls.

### Cell invasion assay

Cells were given serum reduced (0.5% FBS) media for 24 h, then seeded at a density of 40,000 (MDA-MB-231) or 69,000 (MCF7) in the upper chamber of 24 well transwell inserts with 8 μm pores. For MDA-MB-231 cells, inserts (Corning) were rehydrated in cell culture media in a humidified chamber for 2 h, then transferred to a companion plate using sterile forceps. For MCF7 cells, inserts were coated in 0.2 mg/mL growth factor reduced Matrigel Matrix (BD Bioscience) and left to set in a humidified chamber for 2 h. Protocol for the cell migration assay was then followed for both cell lines.

### Cell proliferation assay

BrdU-ELISA assays were used to examine cell proliferation with pharmacological treatment. 10,000 cells were seeded in a 96-well plate and the next day full media was replaced with serum-reduced media for 24 h. Antagonists were applied for 30 min following the addition of agonist or vehicle control in serum-reduced medium. Cells were incubated in normoxia or hypoxia for 20 h followed by an additional 4 h incubation with 1X BrdU substrate (Abcam, ab126556). Cells were then fixed and BrdU incorporation was measured according to manufacturer instructions.

### Spheroid formation and growth assays

10,000 cells were plated in round-bottom U shaped 96-well plates (Corning). The plates were then spun in a circular motion to promote aggregation of cells into a single spheroid per well. Spheroids were then grown for 72 h. Once spheroids reached a size of 1-1.2 mm in diameter, antagonist and agonist treatments were applied. Following 24 h of treatment, a minimum of 24 MDA-MB-231 or 16 MCF7 spheroids were collected per condition for RNA extractions and qPCR analysis. Images of spheroids were captured on a Nikon Eclipse Ti Microscope after treatments at 0 and 24 h. Surface area of spheroid was measured using ImageJ Fiji software to assess spheroid growth. Spheroids were cultured in normoxia, but produce a hypoxic microenvironment that make them valuable tools to study the tumor microenvironment. We detected the degree of spheroid hypoxia by using CAIX mRNA levels as a hypoxia marker in an RT-qPCR of spheroid lysates compared to a normoxic monolayer.

### Spheroid invasion assay

10,000 cells were plated in flat bottomed 96-well plates (Thermo Scientific) coated with 1.5% low melting agarose (Fisher Scientific). MDA-MB-231 spheroids were grown on Spheroid Formation ECM (Cultrex). The plates were spun in a circular motion to promote aggregation of cells in the middle of each well into single spheroids. Spheroids were then grown for 72 h before embedding in vehicle control or antagonist-enriched ECM (Cultrex). After 30 min, agonists were applied on top of solidified ECM in serum-reduced media. Images were captured of spheroids after initial treatment and at intervals of 0 h, 24 h, 48 h, and 96 h to assess treatment impact on invasion into surrounding ECM on a Nikon Eclipse Ti Microscope. The invasive protrusions into the ECM were measured using ImageJ Fiji software.

### Immunofluorescence

Formalin-fixed paraffin-embedded human tumour tissue samples with a thickness of 5 μm were obtained from the Ontario Tumour Bank (46 invasive ductal breast carcinoma cases and 10 adjacent normal breast tissue), which is supported by the Ontario Institute for Cancer Research through funding provided by the Government of Ontario. Samples were chosen based on receptor status such that 24 were negative for the estrogen receptor (ER), progesterone receptor (PR), and the human epidermal growth factor receptor 2 (HER2), and 22 were positive for the estrogen receptor to reflect the cell lines used in this study. Sample pathological T (tumour), grade, clinical M (metastases), and patient outcome were also considered. Samples on microscope slides were rehydrated using xylene and serial dilutions of ethanol followed by a rinse in deionized water and a wash with 1XTBS. Antigen retrieval was then preformed using pre-heated antigen retrieval buffer (10 mM Tris, 1 mM EDTA, 0.05% Tween 20, pH 9.0) at 85 °C for 30 min in a water bath. Slides were permeabilized using TBS + 0.025% Triton X-100 then blocked in 10% goat serum with 1% BSA in TBS for 2 h at room temperature. CAIX at 1/35 (Novus, NBP1-51691) was combined with either NPY1R at 1/100 (Abcam, ab91262) or NPY5R at 1/400 (Abcam, ab133757) at 4 °C overnight. Primary antibody was omitted for the negative controls. Slides were then counterstained with 1/100 Alexa Fluor 555 (Life Technologies) and 1/250 Alexa Fluor 488 (Cell Signaling Technologies) for one hour followed by 1/50,000 Hoechst (Cell Signaling Technologies) for 8 min. Slides were then mounted using ProLong Gold (ThermoScientific) and images were captured on a Nikon Eclipse Ti Microscope. CAIX, NPY1R, and NPY5R expression and colocalization were quantified using ImageJ Fiji software. Deconvolution was performed using the Iterative Deconvolution plugin with 16 iterations followed by colocalization analyses using the JACoP plugin to calculate fractional overlap between CAIX and NPY1R/NPY5R using Manders’ Colocalization Coefficients after thresholding. The same threshold was used to calculate the percent positive pixels for NPY1R, NPY5R, and CAIX. Full and informed patient consent was obtained, and the project was approved by the University of Guelph Research Ethics Board Committee and Ontario Institute for Cancer Research Ethics Committee.

### RNA extraction and qRT-PCR

RNA was extracted using Trizol (Invitrogen) per manufacturer’s instructions. 2 µg of RNA was reverse transcribed using a high-capacity cDNA reverse transcription kit (Applied Biosystems). Primers used are described in Table S1. Quantitative PCR was performed using SsoAdvanced Universal SYBR Green Supermix (BioRad). Data was retrieved with CFX manager software (BioRad) and melting curves were examined to confirm the absence of primer dimers. Relative fold change of expression was calculated using the ΔΔCT method, and transcript levels were normalized to endogenous controls RPLP0 and RPL13A and then compared to internal controls.

### Statistical analysis

Statistical analyses were performed using GraphPad and data are presented as mean ± SEM. The Bartlett test was used to verify the normal distribution of data. Statistical differences between treatments were then evaluated by one-way ANOVA followed by Tukey’s honestly significant difference (HSD) post-hoc test.

## Results

### The MAPK pathway is preferentially repressed in hypoxia by isoform-specific NPYR antagonists

**Fig. 1:**
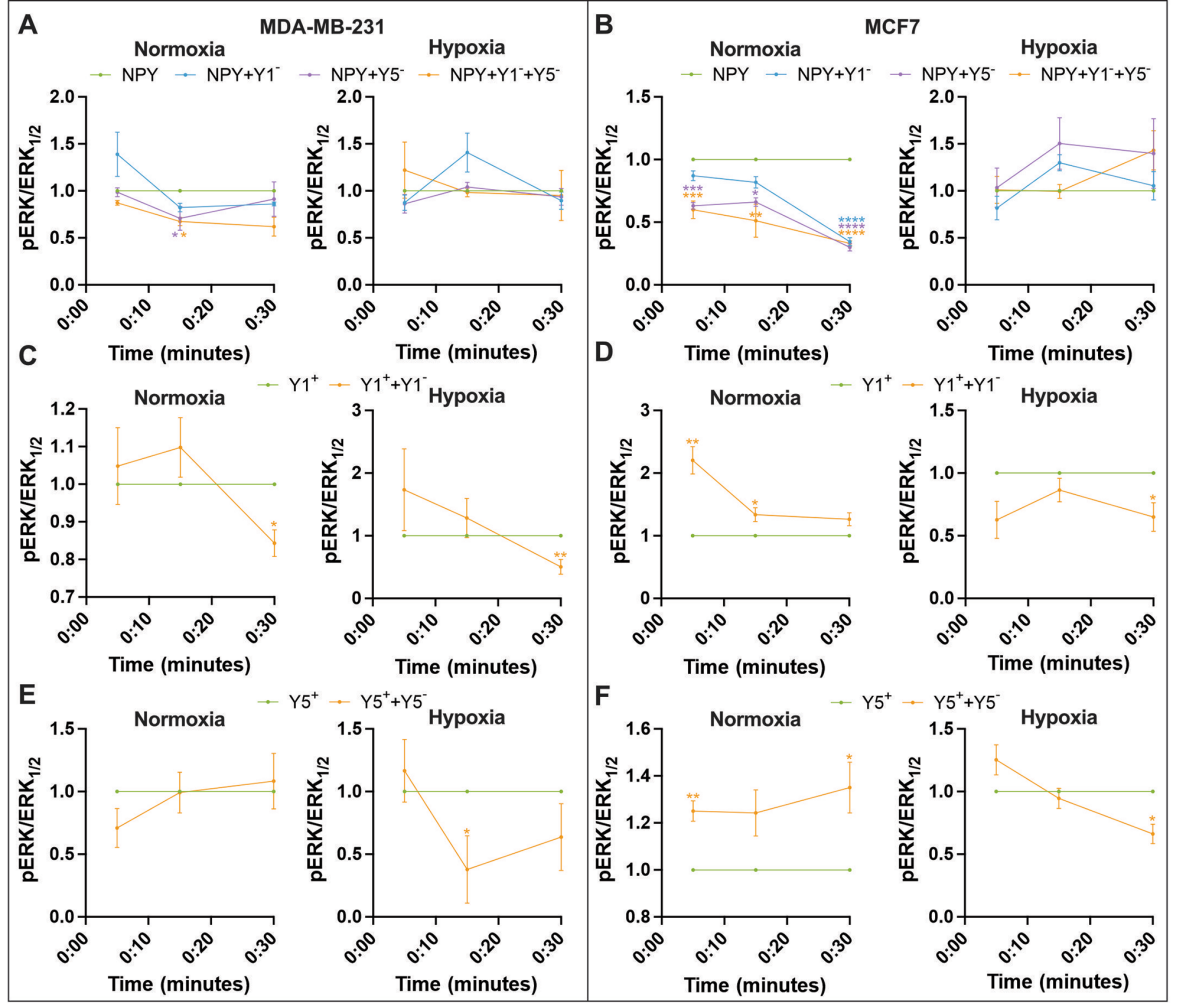
**The MAPK pathway is preferentially repressed in hypoxia by isoform-specific NPYR antagonists**. ERK1/2 phosphorylation was measured in (A, C and E) MDA-MB-231 and (B, D and F) MCF7 cells treated with subtype-specific NPYR antagonists (Y1^−^ or Y5^−^) for 30 min followed by agonist stimulation in normoxia and hypoxia for 5, 15 and 30 min. A general agonist that stimulates all NPYR subtypes (NPY; A and B) or subtype-specific agonists (Y1^+^; C and D, or Y5^+^; E and F) were used. Data (n ≥ 3) represent the mean fluorescence of pERK1/2 after treatment and normalization to total ERK1/2. Each antagonist treatment is made relative to the respective agonist alone at each individual time point. Line graph with error bars representing the SEM and * represent p < 0.05 using either a one-way ANOVA and Tukey’s HSD post-hoc test or an unpaired t-test

MAPK activity contributes to macrobiological processes such as migration, proliferation, and invasion [23]. We previously demonstrated that NPYRs signal through the MAPK pathway, with an earlier peak activity in hypoxic cells compared to normoxic cells [19]. To test whether NPYR antagonists can reduce signaling through the MAPK pathway in hypoxia, we measured the phosphorylation of ERK1/2 over a time course. pERK1/2 was not induced by any agonist in our treatments, likely because this pathway is upregulated at baseline as shown in a large cohort of breast cancers [24] and in cancer cell lines including MDA-MB-231 [25]. However, we could reduce pERK1/2 levels with NPYR antagonist treatment in several conditions when making each antagonist treatment relative to the respective agonist alone at each individual time point. MDA-MB-231 cells treated with NPY displayed 40–50% higher pERK1/2 levels, only in normoxia, after 15 min of stimulation relative to cells receiving the Y5-specific agonist at the same time interval (Fig. 1A). Similarly, only in normoxia did MCF-7 cells treated with NPY display up to 70% more pERK1/2 than cells treated with Y1-specific or Y5-specific antagonists (Fig. 1B). When cells were treated with Y1-specific agonist, both cell lines displayed significant reductions in pERK1/2 after 30 min of Y1-specific antagonist (Fig. 1C-D), but in MCF7 it was only in hypoxia (Fig. 1D). Upon stimulation with Y5-specific agonist, only hypoxic cells displayed a significant reduction in pERK1/2: MDA-MB-231 displayed a 62% decrease in pERK1/2 after 15 min of stimulation (Fig. 1E) and MCF7 displayed a 33% decrease in pERK1/2 after 30 min of stimulation (Fig. 1F). These data suggest that the MAPK pathway can be antagonized by existing NPYR antagonists. Further, hypoxic MDA-MB-231 and MCF7 cells were more vulnerable when NPY1R and NPY5R isoforms were specifically stimulated and antagonized.

### Hypoxia-driven proliferation can be inhibited in MDA-MB-231, but not MCF7 cells

**Fig. 2:**
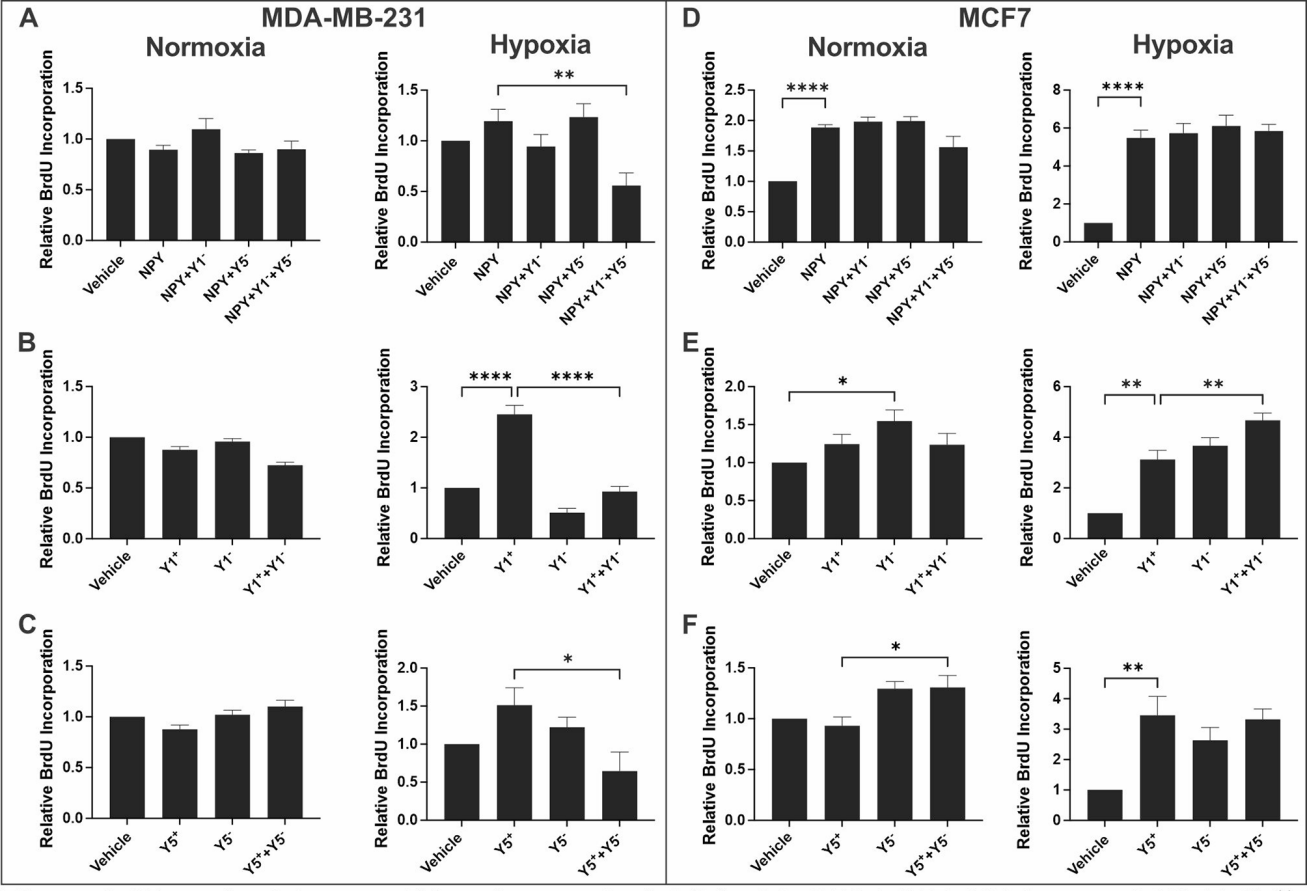
**Hypoxia-driven proliferation can be inhibited in MDA-MB-231 but not MCF7**. Cell proliferation was measured by BrdU incorporation in (**A**-**C**) MDA-MB-231and (**D**-**F**) MCF7 cells treated with subtype-specific NPYR antagonists (Y1^-^ or Y5^-^) following stimulation in normoxia and hypoxia. A general agonist that stimulates all NPYR subtypes (NPY) or subtype-specific agonists (Y1^+^ or Y5^+^) were used. Data (n ≥ 3) represent the mean % BrdU-positive cells after treatment relative to vehicle control. The Bartlett test was used to verify that all data sets were normally distributed. Bar graph with error bars representing the SEM and * represent p < 0.05 using a one-way ANOVA and Tukey’s HSD post-hoc test

Hypoxia enhances NPY-dependent proliferation in MDA-MB-231 and MCF7 cell lines [19], but it is unknown whether this effect can be antagonized. We treated cells with antagonists specific for NPY1R and/or NPY5R before stimulating with agonists and measuring proliferation via BrdU incorporation. Proliferation was only stimulated in hypoxic, but not normoxic, MDA-MB-231 cells with either the general NPY agonist (Fig. 2A) or Y1- or Y5-specific agonists (Fig. 2B-C). This hypoxic stimulation in proliferation was reduced, and in some instances below the basal non-stimulated levels, by 2.16- to 2.6-fold when cells were treated with Y1- and Y5-specific antagonists together (Fig. 2A) or alone (Fig. 2B-C). MCF7 proliferation was induced more potently in hypoxia relative to normoxia, but could not be antagonized with any antagonist treatment (Fig. 2D-F). In some cases, the antagonists alone enhanced proliferation in MCF7. These data suggest that NPY1R and NPY5R-driven proliferation of MDA-MB-231, but not MCF7, can be antagonized more greatly in hypoxia by receptor-specific antagonists.

### NPY-driven cell migration and invasion can be antagonized to a greater extent in hypoxia

**Fig. 3:**
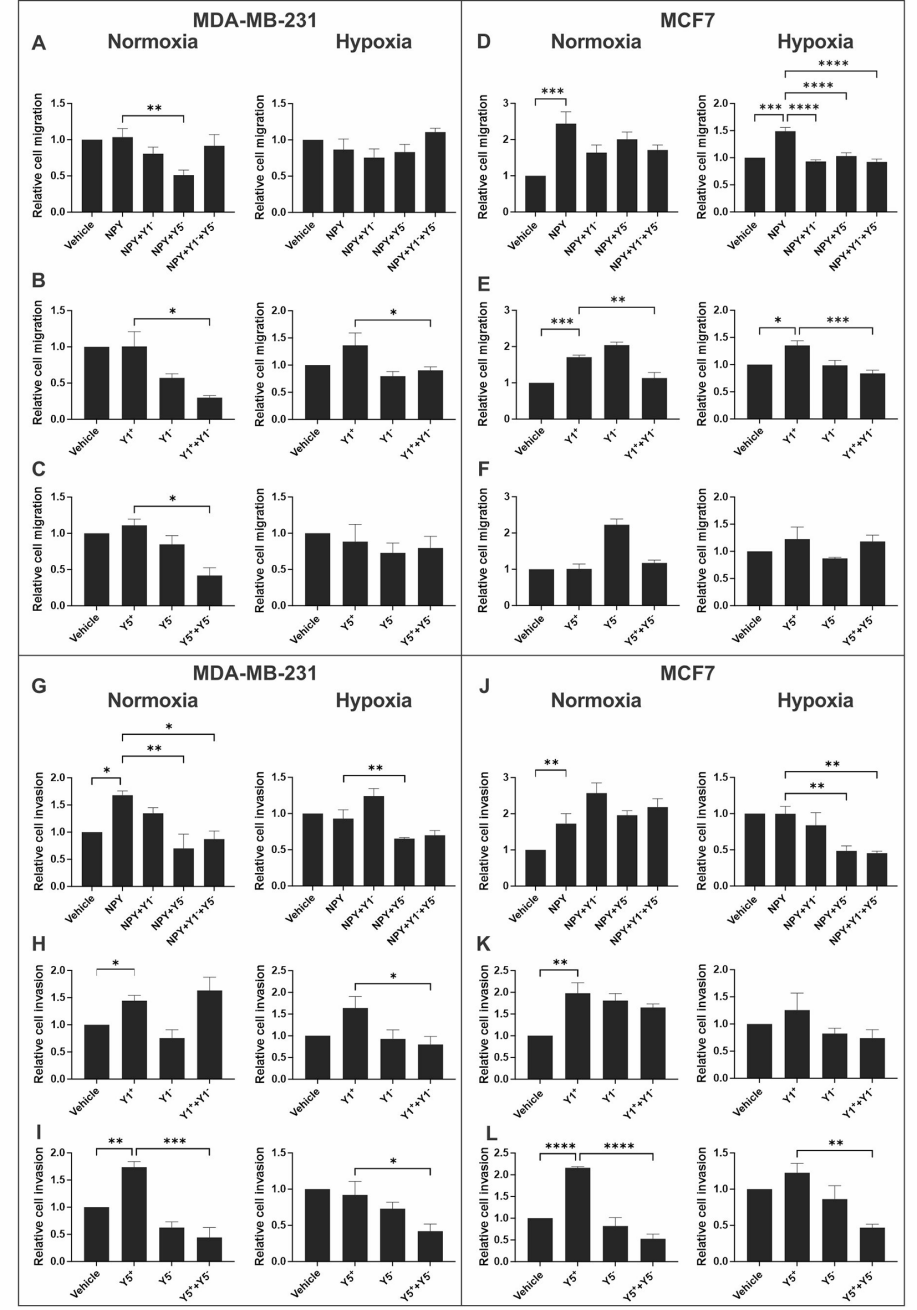
**NPY-driven cell migration and invasion can be antagonized to a greater extent in hypoxia**. Cell migration (**A**-**F**) and invasion (**G**-**L**) were measured via transwell assay (without or with Matrigel-coated inserts) in MDA-MB-231 (**A**-**C** and **G**-**I**) and MCF7 (**D**-**F** and **J**-**L**) cells treated with subtype-specific NPYR antagonists (Y1^-^ or Y5^-^) in normoxia and hypoxia. A general agonist that stimulates all NPYR subtypes (NPY) or subtype-specific agonists (Y1^+^ or Y5^+^) were used. Data (n ≥ 3) represent the number of cells migrated after 22 h (MDA-MB-231) or 24 h (MCF7) relative to vehicle control. The Bartlett test was used to verify that all data sets were normally distributed. Bar graph with error bars representing the SEM and * represent p < 0.05 using a one-way ANOVA and Tukey’s HSD post-hoc test

Cell motility contributes to the establishment of widespread metastases, which makes cancer difficult to treat. We performed transwell migration assays in hypoxic and normoxic MDA-MB-231 and MCF7 cells treated with NPYR isoform-specific antagonists followed by stimulation with agonists. In normoxia, NPY-dependent MDA-MB-231 cell migration was reduced by 2-fold only by the Y5-specific antagonist (Fig. 3A). When normoxic MDA-MB-231 cells were stimulated with Y1-specific or Y5-specific agonists, migration was repressed by 3.46-fold and 2.70-fold, respectively, when a Y1-specific or Y5-specific antagonists were applied (Fig. 3B-C). In contrast, the migration of normoxic MCF7 cells was significantly induced by NPY and Y1-specific agonists but could only be antagonized by the Y1-specific antagonist (Fig. 3D-F). Hypoxia abolished the ability of Y5-dependent migration to be antagonized in MDA-MB-231 cells (Fig. 3C), but sensitized NPY-dependent migration in MCF7 cells to Y1- and Y5-specific antagonists (Fig. 3D).

To further understand the impact of these drug treatments on mechanisms of cancer progression, we investigated cellular invasion via transwell assay with Matrigel. Normoxic invasion of MDA-MB-231 cells was significantly induced by all three agonists (Fig. 3G-I), but could only be antagonized when the Y5-specific antagonist was applied (Fig. 3G and I). The invasion of MDA-MB-231 cells was similarly reduced in hypoxia by Y5-specific antagonist (Fig. 3G and I). However, hypoxia sensitized Y1-dependent MDA-MB-231 cell invasion to Y1-specific antagonist (Fig. 3H). The invasion of MCF7 cells was also significantly induced by all three agonists in normoxia (Fig. 3J-L) and only the Y5-dependent invasion could be reversed by its specific antagonist (Fig. 3L). In MCF7 cells, hypoxia rendered NPY-dependent invasion more sensitive to the Y5-specific antagonist (Fig. 3J). Our data highlight that NPY-driven cell migration and invasion can be reduced with Y1 and Y5 isoform specific antagonists. Hypoxia generally caused the antagonists to have greater success at reducing migration and invasion, especially in MCF7 cells.

### Antagonizing NPY5R reduces MCF7 spheroid growth and invasion

**Fig. 4:**
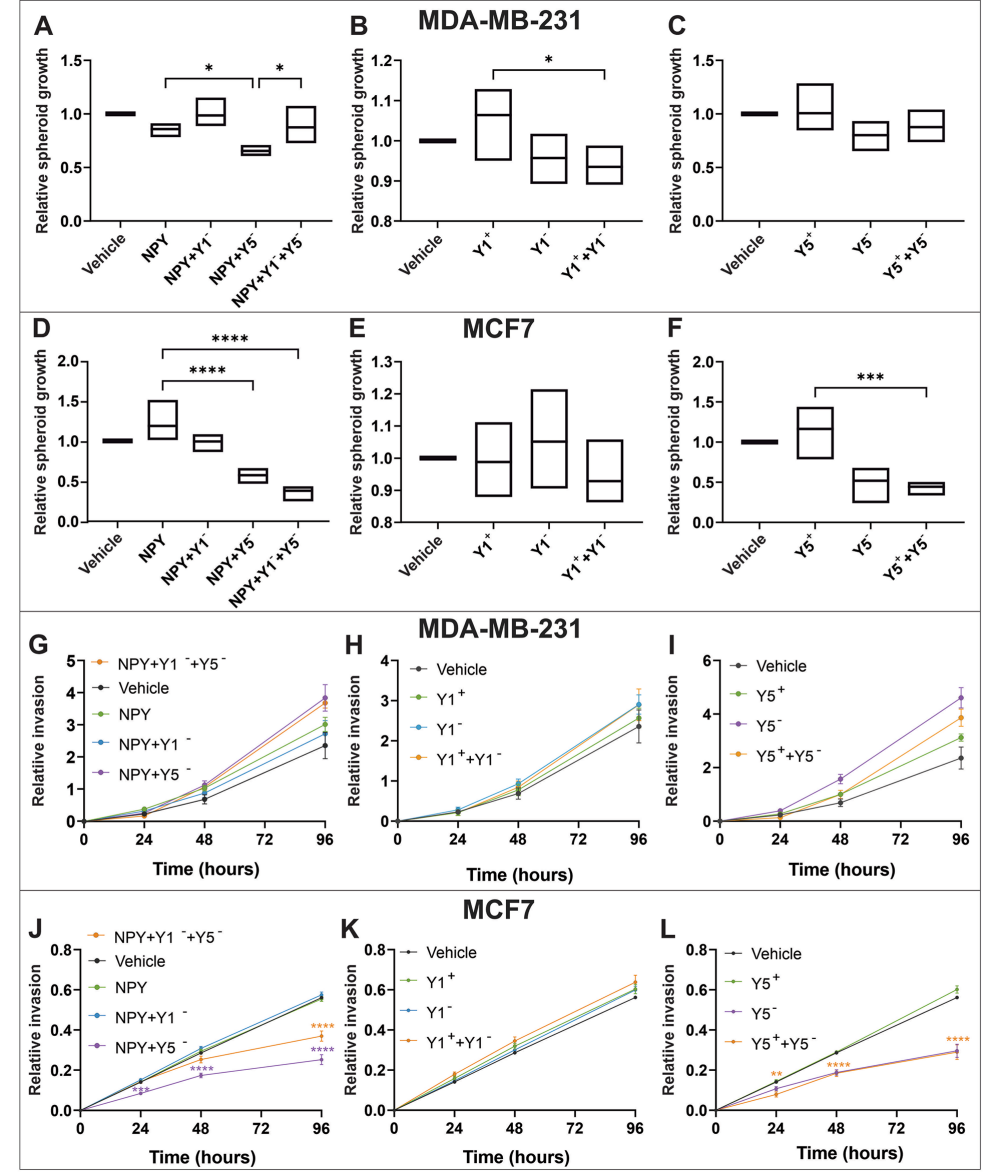
**Antagonizing NPY5R reduces MCF7 spheroid growth and invasion**. Growth of spheroids was measured in (**A**-**C**) MDA-MB-231 and (**D**-**F**) MCF7 cells treated with subtype-specific NPYR antagonists (Y1^-^ or Y5^-^) following stimulation with agonist. Data (n ≥ 4) represent the mean growth of spheroids over 24 h relative to vehicle control. Invasive protrusions of spheroids encased in Matrigel were measured in (**G**-**I**) MDA-MB-231 and (**J**-**L**) MCF7 cells treated with subtype-specific NPYR antagonists (Y1^-^ or Y5^-^) following agonist stimulation over a time course of 96 h. A general agonist that stimulates all NPYR subtypes (NPY) or subtype-specific agonists (Y1^+^ or Y5^+^) were used. All conditions are normalized to their respective vehicle control. The Bartlett test was used to verify that all data sets were normally distributed. Box plots (**A**-**F**) and line graphs (**G**-**L**) have error bars that represent the SEM and * represent p < 0.05 when an antagonist condition was significantly different compared to agonist alone using a one-way ANOVA and Tukey’s HSD post-hoc test

Spheroids are 3D cell culture models that develop necrotic, hypoxic, quiescent, and proliferative zones that are more representative of the tumour microenvironment. We next investigated how NPY agonists and antagonists influence spheroid growth and invasion in the context of a 3D cellular microenvironment. After growing spheroids for three days, we applied the same suite of drug treatments and measured surface area at 0 and 24 h. While none of the agonists stimulated spheroid growth, MDA-MB-231 spheroids treated with NPY and Y5-specific antagonist grew 1.31-fold significantly slower compared to NPY treatment alone (Fig. 4A). While Y5-specific agonist and antagonist did not affect MDA-MB-231 spheroid growth, treatment with Y1-specific agonist and Y1-specific antagonist significantly reduced spheroid growth by 1.14-fold compared to Y1 agonist alone (Fig. 4B-C). In MCF7, inhibition of NPY-dependent spheroid growth with NPYR antagonists was more potent relative to MDA-MB-231. Treating MCF7 spheroids with Y5-specific antagonist reduced their growth by 2.07-fold relative to NPY alone (Fig. 4D). Treating with Y1-specific antagonist did not reduce spheroid growth in MCF7 (Fig. 4E). However, Y5-specific antagonist reduced MCF7 spheroid growth by 2.62-fold relative to Y5-specific agonist alone (Fig. 4F). Our data indicate a modest reduction in MDA-MB-231 spheroid growth, but a significantly greater than 2-fold reduction in MCF7 spheroid growth with the Y5-specific antagonist.

To determine invasion in a 3D context, spheroids were embedded in Spheroid Formation Extracellular Matrix (ECM) and then treated with agonist and/or antagonists. Invasive protrusions were measured at 0, 24, 48, and 96 h post-treatment. We did not observe any significant effects of agonist and/or antagonist treatments on MDA-MB-231 spheroid invasion (Fig. 4G-I). In MCF7, spheroid invasion was significantly impaired by Y5-specific antagonist compared to agonist alone as early as 24 h after treatment, and every time interval afterward. This was observed relative to NPY stimulation (2.19-fold decrease) and Y5-specific stimulation (2.08-fold reduction) (Fig. 4J-L). Interestingly, Y5 antagonist alone (without agonist stimulation) significantly impaired spheroid invasion (Fig. 4L). Overall, NPY-dependent spheroid growth and invasion were more potently impaired by NPYR antagonist treatment in MCF7 compared to MDA-MB-231 primarily through the NPY5R axis.

### NPYR antagonism increases expression of NPY1R and NPY5R, but reduces CAIX levels

**Fig. 5:**
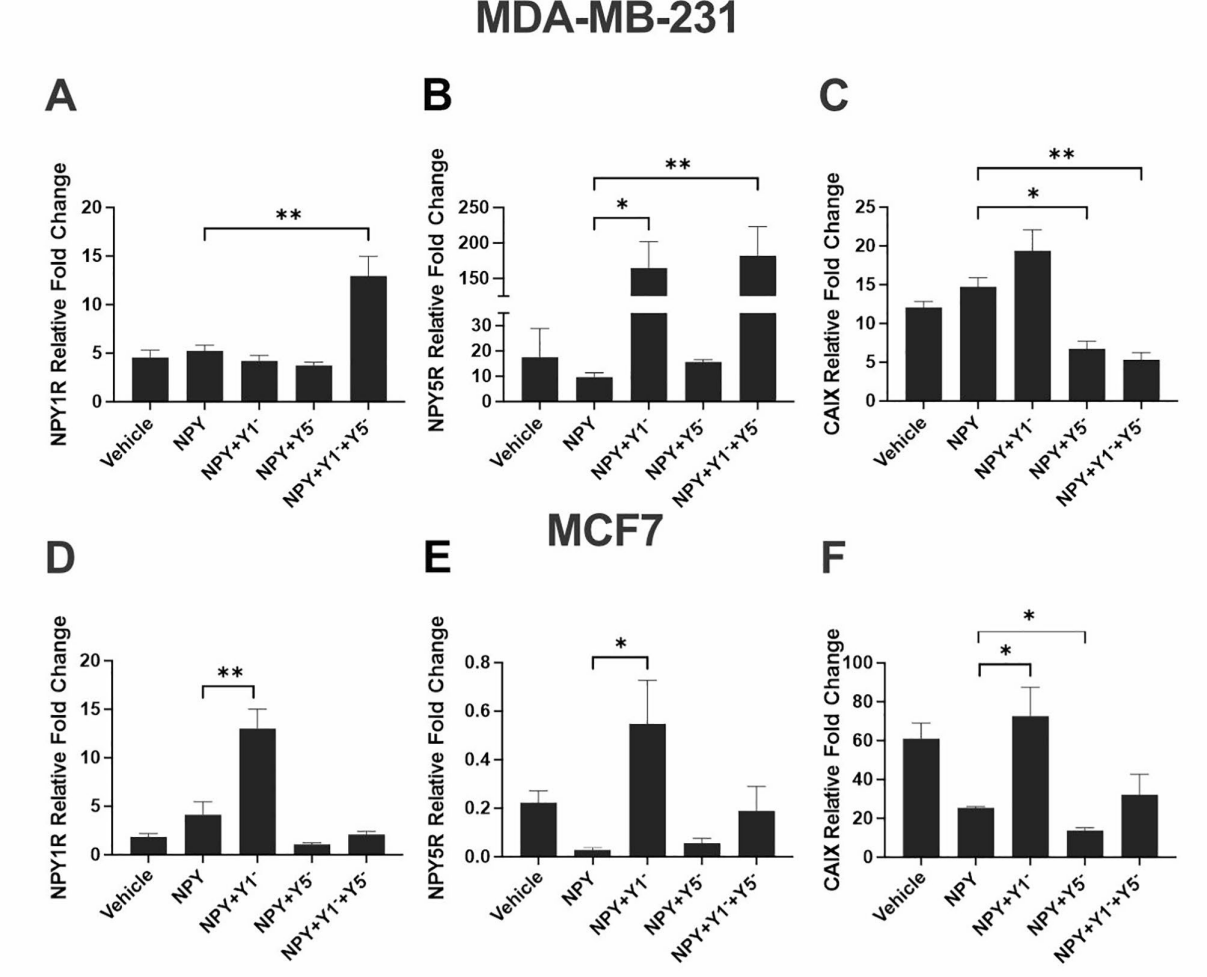
**NPYR antagonism increases expression of NPY1R and NPY5R, but reduces CAIX levels**. Spheroids of MDA-MB-231 (**A**-**C**) and MCF7 (**D**-**F**) breast cancer cells were lysed and NPY1R (**A** and **D**), NPY5R (**B** and **E**), and CAIX (**C** and **F**) mRNA levels were measured by qRT-PCR. Data normalized to endogenous control genes *RPLPO* and *RPL13A* and made relative to normoxic monolayer. Bar graphs with error bars representing the SEM and * represent p < 0.05 using a one-way ANOVA and Tukey’s HSD post-hoc test

We next investigated whether antagonizing the NPYRs could influence NPYR and CAIX mRNA abundance in spheroids. Antagonizing the NPYRs could cause a compensatory feedback loop to increase the expression of the impaired receptor. Since the NPYRs produce a broad signaling cascade, potential impacts to CAIX expression, a hypoxia marker, could be responsible for the reductions in spheroid size and invasion that we observed. We measured mRNA abundance via qRT-PCR in MDA-MB-231 and MCF7 spheroids treated with the general NPY agonist and NPY1R- and/or NPY5R-specific antagonists. To gain insight into the effects of these NPYR antagonists on select gene expression in a more physiologically relevant setting, we performed these experiments in the presence of the general NPY agonist in 3D cell culture models that have a heterogenous cell population with respect to oxygen availability. We found that in NPY-stimulated MDA-MB-231 spheroids, a combination of Y1- and Y5-specific antagonists increased NPY1R mRNA abundance by 2.9-fold compared to NPY treatment alone (Fig. 5A). NPY5R mRNA abundance increased by 16-fold and 18-fold when Y1-specific antagonist alone or in combination with Y5-antagonist, respectively, was applied to NPY-stimulated spheroids (Fig. 5B). The hypoxia marker CAIX decreased in mRNA abundance by 2.2-fold and 2.8-fold when NPY-stimulated MDA-MB-231 spheroids were treated with either Y5 antagonist alone or in combination with Y1 antagonist, respectively (Fig. 5C). In MCF7 spheroids stimulated with NPY, NPY1R and NPY5R mRNA abundance increased by 3.2-fold and 21.6-fold, respectively, when treated with Y1-specific antagonist compared to NPY alone (Fig. 5D-E). CAIX mRNA abundance increased by 2.9-fold, but decreased by 1.9-fold, in spheroids treated with Y1- or Y5-specific antagonist, respectively (Fig. 5F). These data show that there is positive feedback for NPY1R and NPY5R expression when they are antagonized. While the efficacy of the antagonists was not impeded by this feedback in several experiments, their failure in some contexts could be explained by this mechanism. Further, the Y5-specific antagonist caused a decrease in CAIX expression, suggesting that a reduction in the hypoxic fraction in spheroids could at least partially explain the Y5-dependent reduction in spheroid size and invasion (Fig. 4).

### Colocalization of NPY5R with tumour hypoxia correlates with metastasis and differentiation while NPY1R expression correlates with patient outcome and survivability

**Fig. 6:**
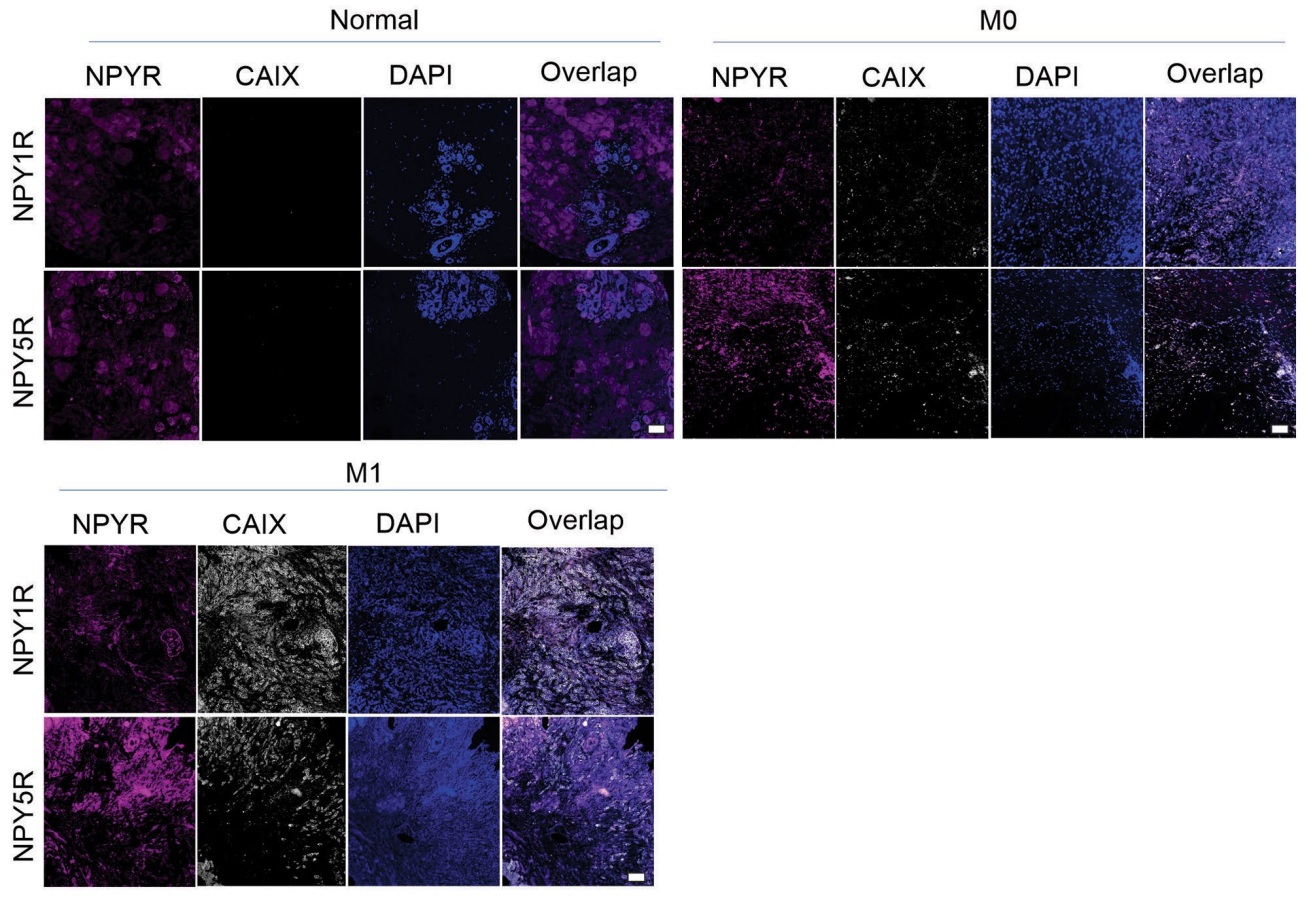
**NPY5R protein levels, but not NPY1R, are higher and colocalize with hypoxia more in metastatic tumors relative to non-metastatic cancer and normal tissue**. Representative immunofluorescence images of human normal (n = 10) and cancer (n = 46) breast tissue sections used in the analyses for Fig. 7. The 46 breast tumor samples were divided into M0 (non-metastatic; n = 35) and M1 (metastatic; n = 11). These breast tumor samples had associated stage, grade, receptor status, and patient outcome information and were also used in the analyses in Fig. 7 and Figure S1. CAIX was used as a marker of hypoxia. DAPI was used as a marker of cells (nuclei). Scale bar, 100 μm

**Fig. 7:**
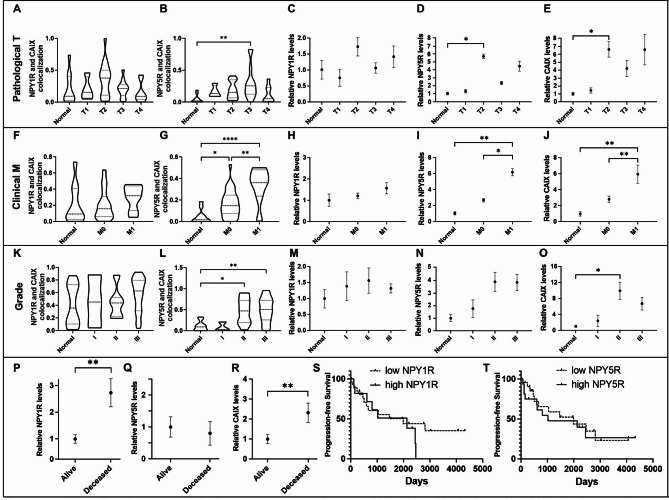
**Colocalization and clinical parameter correlation of NPY1R, NPY5R, and CAIX in human breast carcinomas**. Immunofluorescence of NPY1R, NPY5R, and CAIX (hypoxia marker) was performed on 46 human breast tumor and 10 normal breast tissue sections. A minimum of 4 immunofluorescence images per slide (section) were evaluated using Fiji for ImageJ with the JACOP plugin. Proteins levels were quantified and Manders overlap coefficient was calculated for NPY1R or NPY5R with CAIX. Data was then presented as violin plots based on the patient parameters of (**A**-**E**) Pathological T, (**F**-**J**) Clinical M, (**K**-**O**) and Grade. (**D**) Samples were classified by current patient status (alive or deceased) and correlated to the protein abundance of NPY1R (**P**), NPY5R (**Q**) and CAIX (**R**). Kaplan-Meier plots assessing progression-free survival of breast cancer patients grouped into high and low NPY1R (**S**) or NPY5R (**T**) expression using the median expression between the two groups as a threshold. Error bars represent the SEM. and * represent p < 0.05 using a one-way ANOVA and Tukey’s HSD post-hoc test

Immunofluorescence analyses of breast tumour and normal tissue was conducted to determine correlation between NPY1R or NPY5R protein levels and a marker of hypoxia (CAIX) in breast carcinoma (Fig. 6). Further, patient metrics such as pathological T, clinical M, grade, hormone receptor status, age, and survival were correlated with NPYR protein levels and their overlap with hypoxic regions. NPY1R did not colocalize more with hypoxic tumor regions across cancer stages (Fig. 7A), but NPY5R significantly colocalized with hypoxia in stage T3 tumors by 6.53-fold relative to normal breast tissue (Fig. 7B). Total NPY1R protein did not significantly change between normal and cancer tissue (Fig. 7C), however both NPY5R and CAIX protein were higher in stages 2–4 relative to normal tissue with significant 6-fold increases in stage T2 (Fig. 7D-E). We observed higher colocalization between NPY5R and CAIX (7.16-fold) in metastatic cancer compared to non-metastatic cancer (Fig. 7F-G). Further, there was a higher overall protein expression of NPY5R (2.30-fold) and CAIX (2.12-fold), but not NPY1R, in metastatic cancer relative to non-metastatic cancer (Fig. 7H-J). Tumors with poorly differentiated cells (grades II and III) were more likely to have higher CAIX overlap with NPY5R (4.29-fold and 4.48-fold), but not with NPY1R (Fig. 7K-L). Higher protein levels of NPY5R, but not NPY1R, were observed in poorly differentiated cells (grades II and III) relative to normal tissue (Fig. 7M-N). Significantly higher CAIX protein in moderately differentiated tumor cells (9.81-fold) relative to normal tissue was observed (Fig. 7M-O). We did not observe any correlation between NPY1R and NPY5R protein levels and hormone receptor status or alive/deceased status of the patients (Fig. S1A-C), but there was significant increase in NPY1R, NPY5R, and CAIX in patients aged 84–95 relative to some younger age groups (Fig. S1D). These data suggest that NPY5R protein levels and colocalization with hypoxic tumor regions correlate with cancer stage, metastatic potential, and abnormal tumor cells.

Conversely, NPY1R protein abundance was found to be a predictor of survival. A significantly higher proportion of samples belonging to currently deceased patients had higher NPY1R and CAIX protein, but not NPY5R, compared to patients who are alive at the time of this study (Fig. 7P-R). Moreover, when patient samples were grouped into high and low NPY1R or NPY5R based on the median there was a 0% probability of progression-free survival after 2466 days in the high NPY1R group while patients with low NPY1R had a 44.08% progression-free survival after 2766 days (Fig. 7S). NPY5R levels did not affect the probability of progression-free survival (Fig. 7T). When cancer specimens were grouped by receptor status (ER + or TNBC), the high NPY1R group still displayed lower progression-free survival in both cancer subtypes compared to the low NPY1R group (Fig. S1E). NPY5R levels were not a predictor of lower progression-free survival in either cancer subtype (Fig. S1F). These data indicate that both NPY1R and NPY5R expression and/or colocalization with tumor hypoxia are linked to markers of tumor progression and survival.

## Discussion

NPY1R and NPY5R first gained relevance in breast cancer research because of their high abundance and density compared with all other NPYR-positive tumors [9]. This characteristic has been exploited to develop chemically modified analogs of NPY that are used in breast cancer imaging and diagnosis [10–12]. We investigated whether such analogs could antagonize NPY1R and NPY5R to influence cancer hallmarks in two different breast cancer cell lines while considering the effects of hypoxia, a major component of the tumor microenvironment. We found that, in general, antagonizing NPY1R and/or NPY5R in hypoxia can more greatly reduce MAPK signaling, cell proliferation, cell migration and invasion, and spheroid growth and invasion. This suggests that inhibiting the NPYRs could be a clinically relevant avenue. However, antagonizing either NPY1R or NPY5R often produced different effects with respect to oxygen or cell line. This reveals that NPYR signaling and function in breast cancer has complexities that need to be discussed and researched further.

We noticed during the MAPK signaling assay that pERK1/2 was not induced by any agonist in any of our treatments, even though such activation was observed in other cell lines [3, 4, 26]. The MAPK pathway can be constitutively active in breast cancer [24] and in some cell lines such as MDA-MB-231 [25], and it is possible that a higher baseline pERK1/2 is difficult to induce further in these cell lines. We would like to note that a previous study from our group could produce an induction of pERK1/2 with NPY. The MCF7 and MDA-MB-231 cells were obtained from the ATCC in both studies but at different times. This could point to genomic variability and clonal evolution that can be observed within cell lines. A thorough analysis of this phenomenon was done in HeLa [27], but has also been observed in MCF7 and MDA-MB-231 [28]. These cell lines in both studies display many consistencies such as their agonist-induced proliferation that is more potent in hypoxia (Fig. 2). Even with what appears to be higher baseline levels of pERK1/2, we were still able to repress pERK1/2 levels with NPY1R and NPY5R antagonists in some contexts (Fig. 1). We observed repression of pERK1/2 in normoxia in both cell lines, but this effect was negated by hypoxia (Fig. 1A-B). Hypoxia may influence NPY agonist efficiency through crosstalk between NPY1R and NPY5R that includes heterodimerization and receptor recycling changes [29, 30]. When we used Y1- and Y5-specific agonists, we observed almost no reductions in pERK1/2 levels in normoxia. In hypoxia, however, both isoform-specific agonists did reduce pERK1/2 levels in all conditions. Isoform-specific NPY agonists are physiologically relevant. For example, there is a hypoxia-induced peptidase DPPIV that cleaves the general NPY agonist into a Y5-specific agonist [31]. Therefore, hypoxic tumor cells could be more vulnerable to MAPK repression through NPYR antagonists.

Cell proliferation could not be inhibited in MCF7 by NPYR antagonists, while it was inhibited in MDA-MB-231 cells only in hypoxia (Fig. 2). Stimulation of estrogen receptor-ɑ (ER-ɑ) via estrogen has been shown to result in upregulated NPY1R expression in MCF7 cells, but not in MDA-MB-231 cell [32]. Estrogen treatment increases cell proliferation, which can be reduced by addition of NPY. This NPY-induced inhibition of the proliferative effect of estrogen can be rescued by the addition of NPY1R inhibitors [32]. Further, estrogen treatment decreased NPY secretion via the PI3K and AMPK pathways [33]. Due to the evidence of crosstalk between ER-ɑ and the NPYR pathway, we may have stimulated proliferation with NPY agonists via ER-α that could not be reversed with NPYR antagonists. In fact, we produced even more proliferation in MCF7 cells treated with both Y1-specific agonist and antagonist (Fig. 2E). Therefore, the reductions in cell proliferation by NPYR antagonists observed in a triple negative cell line such as MDA-MB-231 cells may not translate to ER-positive cancers.

Similar to the observations for MAPK signaling, repression of cell migration in normoxia by the NPYR antagonists was largely reversed by hypoxia except when the Y1 isoforms were specifically stimulated (Fig. 3). Our data suggest that NPYR antagonists could be more successful at repressing cell migration in hypoxic ER + tumors since NPY-induced MCF7 cell migration was only impaired with antagonists in hypoxia (Fig. 3D). Cell invasion was more uniformly repressed by NPYR antagonists despite cell line differences or oxygen availability. This suggests that the NPYRs could induce more invasion-related genes than migration.

We investigated how the NPYR antagonists influence CAIX expression as a possible explanation for the observed reduction in spheroid growth and invasion. CAIX was used as a marker of hypoxia, but it has also been connected to hypoxic invasion through its interactome in breast cancer cells. CAIX associates with α2β1 integrin, CD98hc, and MMP14 at the leading edge where it donates hydrogen ions required for MMP14 catalytic activity and subsequent extracellular degradation [34]. Indeed, CAIX mRNA levels were reduced only in the presence of the Y5-specific antagonist (Fig. 5C and F). This reduction of CAIX expression could explain the slower spheroid growth and impaired invasion in MCF7 spheroids. MDA-MB-231 spheroids did display some reduction in growth in a Y5-specific manner when stimulated with the general NPY agonist, but invasion was not affected. This could be due to their decreased sensitivity to drugs or the hyperactivity of select pathways discussed earlier. Importantly, we noticed that the antagonists alone sometimes elicited a response or that the antagonists induced the mRNA abundance of the receptors they were antagonizing. This was not entirely surprising since antagonizing one NPYR isoform could influence its dimerization with another isoform or another GPCR altogether. This highlights the importance of performing an antagonist alone control. We did not notice many instances of the antagonist alone eliciting a response, and in fact this control allowed us to reveal a positive feedback loop whereby the antagonist induced the expression of its own receptor or that of another NPYR isoform.

Translating molecular profiles into clinical relevancy is important for bridging the gap between in vitro experiments and patients. In breast tumor tissue compared to normal samples, we show that high NPY5R levels correlate with advanced stage cancer, metastasis, and poorly differentiated cells. Further, higher NPY1R levels correlated with poor patient outcomes such as death and progression-free survival. These data are in agreement with the in vitro experiments showing that spheroid growth and invasion flow mostly through the NPY5R axis. However, it is unclear why NPY1R abundance is more strongly connected to outcome, which currently limits the clinical relevance of NPY5R expression levels in breast cancer.

Research into the complexities of how the NPYR isoforms interact with one another and perhaps other GPCRs in normoxia and hypoxia to influence signaling cascades is still in its infancy. This study highlights that the development of NPYR antagonists in breast cancer therapy and patient-based treatment plans could be a promising avenue to continue pursuing.

## Electronic supplementary material

Below is the link to the electronic supplementary material.

Supplementary Material 1

## Data Availability

The datasets used and/or analysed during the current study are available from the corresponding author on reasonable request.
