# Supplementary material for: Pharmacological inhibition of neuropeptide Y receptors Y1 and Y5 reduces hypoxic breast cancer migration, proliferation, and signaling
Source: BMC Cancer. 2023 Jun 1;23:494. doi: 10.1186/s12885-023-10993-1 (PMC10234023; doi:10.1186/s12885-023-10993-1)
Supplement: Supplementary file 1 — Supplementary Material 1 [file 12885_2023_10993_MOESM1_ESM.pdf]

Table S1: Primers used for qPCR.

Target	Forward 5’ to 3’	Reverse 5’ to 3’
NPY1R	CCATCGGACTCTCATAGGTTGTC	GACCTGTACTTATTGTCTCTCATC
NPY5R	CCTCAGGTGAAACTCTCTGGCA	GAGAAGGTCTTTCTGGAGCAGG
RPLP0	AACATCTCCCCCTTCTCC	CCAGGAAGCGAGAATGC
RPL13A	AGCCGCATCTTCTGGCGGA	TTGTCGTAGGGCGGTGGGATG
C-FOS	ATACACTCCAAGCGGAGACAG	TCCTTCTCCTTCAGCAGGTTG
CCND1	GTGCCACAGATGTGAAGTTCATT	CTCTGGAGAGGAAGCGTGTG
CAIX	TAAGCAGCTCCACACCCTCT	CCTCAATCACTCGCCCATTCA

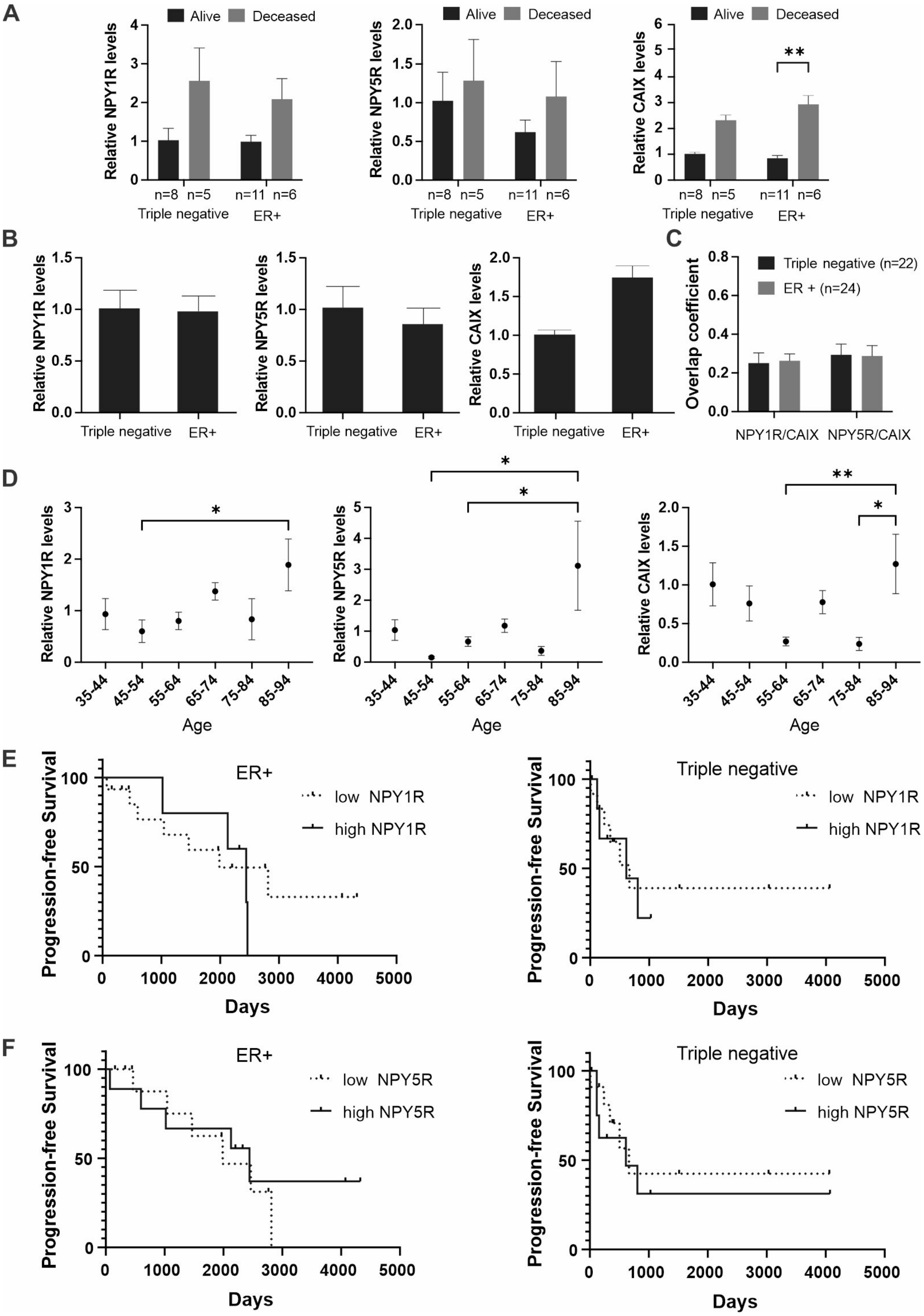

A
Alive
Deceased
Relative NPY1R levels
Relative NPY5R levels
Relative CAIX levels
**
n=8 n=5 n=11 n=6
Triple negative
ER+
B
C
Triple negative (n=22)
ER + (n=24)
Overlap coefficient
NPY1R/CAIX
NPY5R/CAIX
D
*
**
35-44
45-54
55-64
65-74
75-84
85-94
Age
E
ER+
low NPY1R
high NPY1R
Progression-free Survival
Days
Triple negative
F
low NPY5R
high NPY5R

Figure S1. Correlation of NPY1R, NPY5R, and CAIX protein levels with alive/deceased status, hormone receptor status and age in patient breast carcinoma tissue samples via immunofluorescence. (A) Relative NPY1R, NPY5R, and CAIX protein levels in breast carcinoma samples grouped by hormone receptor status (triple negative (n=22) and estrogen receptor positive (n=24)) and then each group divided into the alive or deceased status of the patient. (B) Relative NPY1R, NPY5R, and CAIX protein levels in breast carcinoma samples grouped by hormone receptor status (triple negative (n=22) and estrogen receptor positive (n=24)). (C) Mander's overlap coefficient for the colocalization between NPY1R or NPY5R with CAIX (hypoxia) in triple negative and estrogen receptor positive breast carcinoma samples. (D) Samples were grouped by patient age ranges (25-34; n=1, 35-44; n=4, 45-54; n=9, 55-64; n=14, 65-74; n=5, 75-84; n=6, 85-94; n=7) and age of the patients was correlated to NPY1R, NPY5R, and CAIX protein level. (E-F) Kaplan-Meier plots assessing progression-free survival of breast cancer patients grouped by hormone receptor status (triple negative (n=22) and estrogen receptor positive (n=24)) and then subsequently divided into high and low NPY1R (E) or NPY5R (F) expression using the median expression as a threshold. A minimum of 4 immunofluorescence images per slide were analyzed using Fiji for ImageJ with the JACOP plugin. Error bars represent the SEM. and * represents $p<0.05$ using a one-way ANOVA and Tukey's HSD post-hoc test.
